# Exploiting deterministic features in apparently stochastic data

**DOI:** 10.1038/s41598-022-23212-x

**Published:** 2022-11-18

**Authors:** Ruedi Stoop, Giuseppe Orlando, Michele Bufalo, Fabio Della Rossa

**Affiliations:** 1 Neuroinformatics and Physics Departments, University and ETH of Zürich, Winterthurerstr. 390, 8057 Zürich, Switzerland; 2 Department of Economics and Finance, Università degli Studi di Bari Aldo Moro, Via C. Rosalba 53, Bari, 70124 Italy; 3 Department of Methods and Models for Economics, Territory and Finance, Università degli Studi di Roma ”La Sapienza”, Via del Castro Laurenziano 9, Rome, 00185 Italy; 4 Department of Electronics, Information and Bioengineering, Politecnico di Milano, Piazza Leonardo da Vinci 32, Milan, 20133 Italy

**Keywords:** Neuroscience, Mathematics and computing, Physics

## Abstract

Many processes in nature are the result of many coupled individual subsystems (like population dynamics or neurosystems). Not always such systems exhibit simple stable behaviors that in the past science has mostly focused on. Often, these systems are characterized by bursts of seemingly stochastic activity, interrupted by quieter periods. The hypothesis is that the presence of a strong deterministic ingredient is often obscured by the stochastic features. We test this by modeling classically stochastic considered real-world data from both, the stochastic as well as the deterministic approaches to find that the deterministic approach’s results level with those from the stochastic side. Moreover, the deterministic approach is shown to reveal the full dynamical systems landscape, which can be exploited for steering the dynamics into a desired regime.

## Introduction

Complexity often emerges from systems composed of many interacting subsystems. Under suitable conditions, such systems produce interesting coherent collective behavior, some of which even evidence universal features. Examples range from classical physics spin systems at criticality^1^, to environmentally relevant biological systems^2,3^ at criticality, and neuronal systems that may or may not be at criticality^4^. All have in common that their dynamics projects down to much lower-dimensional spaces, and that usually periods of fierce activity interchange with calmer periods. Corporate dynamics emerge from economic agents that exchange signals based on prices^5^, which puts it into the class described.

The typical interchange between activity and calmer periods that we find in these systems is, in particular, found in biological neuronal activity. This is one of the reasons why in a recent work^6^, stock market indices developments were predicted by a deterministic low-dimensional neuroinformatics-borrowed Rulkov-type map^7^, and compared to the standard stochastic approach. This comparison yielded slightly superior modeling results of the deterministic over the stochastic one. The crucial observation, however, is that while the generally used stochastic approach fails to offer a convenient understanding of the modeling system’s build-up and of the involved parameters, the Rulkov map approach, due to its simplicity and explicitness, promised to open a door for a deeper understanding of the drivers of the market dynamics—in particular, if a mapping of the real-world data to model parameters could be established^6^.

In the present work, we substantiate these observations and expectations. Here, to emphasize the broad system class embraced by our modeling methodology, we focus on a more general system level and use distinct real-world data. Using data from the market competition between companies as our example, we provide the existence of a convenient handle for guiding such apparently stochastic systems. Competition is an ubiquitous feature of physical and biological systems. For modeling, more specifically, activity developments of firms and the diffusion of innovations in business, Bass-type models^8^ were used (and later extended with behavioural assumptions on forecasting, e.g., Refs.^9,10^). Recently, agent communication and interaction structures have made these processes increasingly neuronal-like^11^. Since also the collective behavior of neuronal ensembles can collectively be modeled by suitably chosen individual neurons^12^, we model the specific real-world data from corporate dynamics by generalizing the Rulkov maps originally designed to reflect the behavior of individual neurons. The results of this modeling are then compared to those obtained from a stochastic ARIMA-EGARCH model (an approach specifically optimized for dealing with autoregressive, moving average, heteroscedastic asymmetric volatility processes). The comparison will evidence that the two approaches are toe-to-toe. The main aim of the present work is, however, to explicitly demonstrate that the simplicity and explanatory power inherent in deterministic models hosts salient advantages over the stochastic approach and to present a perspective of the insight and opportunities available by capitalizing on this.

The present work is made self-sufficient by first repeating the main elements of our deterministic modeling approach. Analytical investigations of our model will reveal in “Stationary states and their stability” section an asymptotic stable stationary state. The precise nature of this stationary state and non-local properties are analyzed in “Numerical investigations of the theoretical model” section using numerical investigations. In the “Android’s market position” section, we will give a wrap-up of how the Android computer operating system achieved a leading position in the market, and what properties the current business state has, according to our analysis. In the conclusion section, we will emphasize the generic nature of the analyzed system type and of the obtained insights.

## Modeling fundamentals

At any level of abstraction of multicomponent systems, increased ’activity’, ’advantage’, or ’profit’ of a subsystem compared to a concurrent subsystem, are the consequences of several factors, such as skills, strategic positioning, etc., and it generally holds that above or below norm profits, respectively, may persist for prolonged periods of time^13–17^, but must converge to zero under perfect competition conditions, in the long run. For the following, we assume that all of these determining factors are combined in a variable that we shall call ’effort’ *e*, a quantity that would not be directly measurable, but manifest itself by consequences: The effort of a system at time $t+1$, $e_{t+1}$, is assumed to depend on a variable *x* characterizing ”profits” according to a bounded functional form

1 $$\begin{aligned} e_{t+1}=\dfrac{\tanh (x_{t})+a}{b}, \end{aligned}$$

which expresses that at $x_{t}=0$, the effort $e_{t+1}$ would be equal to *a*/*b*, but tend to $(a+1)/b$ for $x_{t} \rightarrow \infty$ and to $(a-1)/b$ for $x_{t} \rightarrow -\infty$. To obtain an effort bounded between 0 and 1, it is sufficient to set the parameters equal to $a=1$ and $b=2$. These assumptions entrain a number of consequences. A drop in profit at time *t* puts pressure on the system, forcing it to increase effort. The latter cannot always be maximal, because efforts imply a rise in costs; if profits are high, the firm has to throttle back. As a consequence, we may expect a differential relationship between effort and change in profits of the form

2 $$\begin{aligned} f_n(x_t) = \dfrac{1}{(1+x_t^n)} \, , \qquad n \in {\mathbb {N}},\;x\in {\mathbb {R}}. \end{aligned}$$

As an illustration, in the business context, a strong link between demand, hours worked, and the profits can be expected. Whereas econometric analysis has not yet revealed the precise form of this link, previous work^18^ suggests that profits are mainly correlated with average working hours, i.e., is expressed by the concept of effort by Eq. (1), and that the function $f_n(x)$ defined defined in (2) has the following properties

### Proposition 2.1

For *n* even

(i) $$f_n$$ is bounded: $$0<f_n(x)\le 1(=f_n(0))$$,
(ii) $f_n(x)$ vanishes for $x \rightarrow \pm \infty$,
(iii) the first derivative of $f_n(x)$ is bounded. More specifically, $\max _{x\in {\mathbb {R}}}|f'_n(x)|< \frac{n}{2}$.

### Proof

(i), (ii) are obvious.

(iii) Observe that $f'_{n}(x)=-\frac{n x^{n-1}}{(1+x^n)^2}$ and set $g(x): =-\frac{f'_n(x)}{n}$, from where we obtain $g'(x)=\frac{x^{n-2}}{(1+x^n)^3}[(n-1)-(n+1)x^n]$. Moreover, $g'(x)\ge 0 \iff -\root n \of {\frac{n-1}{n+1}} \le x \le \root n \of {\frac{n-1}{n+1}}$, so that *g*(*x*) assumes its global maximum at $x=\root n \of {\frac{n-1}{n+1}}$ and its global minimum at $x=-\root n \of {\frac{n-1}{n+1}}$. Since *g*(*x*) is an odd function, it is easy to see that $\max _{x\in {\mathbb {R}}} |g(x)|= g \left( \root n \of {\frac{n-1}{n+1}} \right) = \frac{n^2-1}{4n^2} \root n \of {\frac{n+1}{n-1}}<\frac{1}{4} \root n \of {\frac{n+1}{n-1}}$. The sequence $\bigl ( \frac{n+1}{n-1}\bigr )_{n\ge 2}$ is decreasing; hence, for all $n\ge 2$ one has $\frac{n+1}{n-1} \le \frac{2+1}{2-1} =3$ and therefore $\root n \of {\frac{n+1}{n-1}}\le \root n \of {3}\le \sqrt{3}<2$.

This proves that $\max _{x\in {\mathbb {R}}}|g(x)|<\frac{2}{4}=\frac{1}{2}\;\; \text {and} \max _{x\in {\mathbb {R}}} |f'_{n}(x)|=n\cdot \max _{x\in {\mathbb {R}}}|g(x)|<\frac{n}{2}$.

$$\square$$

Chosen in this way, *n* monitors the reactivity of profit $x_t$, where lower values provide higher variability. To model company market competition, we will use a generalized version of Rulkov’s map approach developed for describing neuronal dynamics^7^ that takes care of the ”dragging” effect of economic variables that in classical stochastic models is implemented via autoregression^19^: While the change in profit $x_{t+1}$ is the consequence of the previous value $x_t$ and that of a long-trend value $y_{t}$, the value of $y_{t+1}$ depends on its previous value $y_{t}$ and the short-term change $x_{t}$. This can be to first order be implemented by

3 $$\begin{aligned} y_{t+1} = \beta \, y_t - \mu \, x_{t} + \eta , \end{aligned}$$

where $\beta$ represents the sensitivity on the previous value and $-\mu$ is the ’mean reversion’ parameter. Low changes in profit will require high effort, and high efforts predict the recovery of profit. We may schematically depict this dependence by

$$\begin{aligned} \downarrow x_t \, \Rightarrow \, \uparrow e_{t+1} \, \Rightarrow \,\uparrow x_{t+2} \, \Rightarrow \, \downarrow \, e_{t+3} \ldots : \end{aligned}$$

to a high value of $x_t$, a low value of $x_{t+1}$ follows, etc. To smoothen this process, the long-term trend *y* is added

4 $$\begin{aligned} x_{t+1} =\alpha f_n(x_t) + \gamma \, y_t + \delta \, . \end{aligned}$$

Under ’normal’ company performance, the effort will be intermediate, with $x_{t+1}\approx 0$. By combining Eq. (4) with Eq. (3), we obtain

5 $$\begin{aligned} {\left\{ \begin{array}{ll} x_{t+1} = \alpha f_n(x_t) + \gamma \, y_t + \delta \\ y_{t+1} = \beta \, y_t - \mu \, x_{t} + \eta . \end{array}\right. } \end{aligned}$$

In the case of $\gamma$ and $\delta$ equal to zero, the maximum of $f_n(x_t)$ determines the maximum effort. $x_{t+1}$ will reach its peak value at $x_{t}=0$; a small deviation from zero even will cause a dramatic change in the company’s effort. Proposition 2.1 shows that, in the case of $\gamma$ and $\delta$ equal to zero, the maximum of *x* will be $\alpha$.

## Stationary states and their stability

For assessing the influence of *n* in $f_{n}(x_t)$ regarding the stability of Eq. (5), we use some classical results of two-dimensional discrete dynamical systems

6 $$\begin{aligned} {\left\{ \begin{array}{ll} x_{t+1}=F(x_t,y_t) \\ y_{t+1}=G(x_t,y_t), \end{array}\right. } \end{aligned}$$

where *F*, *G* on ${\mathbb {R}}^2\mapsto {\mathbb {R}}$ are any nonlinear functions. A stationary point $(x^*,y^*)$ of Eq. (6) satisfies

$$\begin{aligned} {\left\{ \begin{array}{ll} x^*=F(x^*,y^*) \\ y^*=G(x^*,y^*). \end{array}\right. } \end{aligned}$$

### Lemma 3.1

Consider the quadratic equation $\lambda ^2-b\lambda +c=0$, with $b,c\in {\mathbb {R}}$. Assume that

7 $$\begin{aligned} c<1, \qquad 1-b+c>0, \qquad 1+b+c>0; \end{aligned}$$

then one has $|\lambda _{1,2}|<1$, where $\lambda _{1,2}$ denotes the roots of the quadratic equation.

### Proposition 3.2

Let $(x^*,y^*)$ be a stationary point of system (6) and let *J* be the Jacobian matrix at $(x^*,y^*)$, i.e.,

$$\begin{aligned} J= \begin{bmatrix} F_x(x^*,y^*) &{} F_y(x^*,y^*) \\ G_x(x^*,y^*) &{} G_y(x^*,y^*) \end{bmatrix} \, . \end{aligned}$$

Then $(x^*,y^*)$ is stable if the solutions $\lambda _{1,2}$ of the characteristic equation of *J*

8 $$\begin{aligned} \lambda ^2-Tr(J)\lambda +\det (J)=0 \end{aligned}$$

have modulus smaller than 1, i.e. by Lemma 3.1, if

9 $$\begin{aligned} \det (J)<1, \qquad 1\pm Tr(J)+\det (J)>0. \end{aligned}$$

For deeper insights see, e.g., [27, Chapter V].

### Case $2\le n <+\infty$

#### Theorem 3.3

For $\beta \not =1$, system (5) admits for any even *n* the stationary points

10 $$\begin{aligned} \biggl ( x^*,\frac{\mu x^*-\eta }{\beta -1}\biggr ) \end{aligned}$$

where $x^*$ is any solution of the equation

11 $$\begin{aligned} a x(x^n+1)+ b x^n+d=0, \end{aligned}$$

where

12 $$\begin{aligned} a=\gamma \mu -\beta +1,\;\;b=(\beta -1)\delta -\gamma \eta , \;\;d=(\beta -1)(\alpha +\delta )-\gamma \eta . \end{aligned}$$

Moreover, any stationary point of system (5) is stable if

13 $$\begin{aligned} 0<\alpha<\frac{2}{n}, \qquad 0<\gamma \mu<1, \qquad 0<\beta <1-\gamma \mu . \end{aligned}$$

#### Proof

Stationary points of (5) satisfy

14 $$\begin{aligned} {\left\{ \begin{array}{ll} x (1+x^n)=\alpha +(1+x^n)(\gamma y+\delta ) \\ y=\beta y-\mu x+\eta , \end{array}\right. } \end{aligned}$$

which yields (10,11) as a solution.

It remains to check the stability of the stationary points, i.e. that the Jacobian matrix of system (5)

15 $$\begin{aligned} J(x,y) = \begin{bmatrix} \alpha f'_n(x) &{} \gamma \\ -\mu &{} \beta \end{bmatrix} \end{aligned}$$

satisfies the conditions (9). From (13) and Proposition 2.1 (iii), we have $|\alpha f'_n(x^*)|<\frac{\alpha n}{2}< 1, \;\text {i.e.,} \; -1< \alpha f'_n(x^*)<1.$ With (13), it follows that:

- $$\det \,J=\alpha \beta f'_n(x^*)+\gamma \mu<\beta +\gamma \mu <1,$$
- $$1-Tr\,J+\det \,J=(1-\beta )\bigl ( 1- \alpha f'_n(x^*) \bigr )+\gamma \mu >0,$$
- $$1+Tr\,J+\det \,J=(1+\beta )\bigl ( 1+ \alpha f'_n(x^*) \bigr )+\gamma \mu >0,$$

which concludes the proof. $\square$

These results predict the existence and properties of stationary points of the market competition process as follows. We will start with two explicit examples based on simplifying parameters and finite even *n*, before we turn to the asymptotic case $n \rightarrow \infty$.

#### Example 3.4

Let $n=2$ and $\beta \not =1$. In this case, Eq. (11) reduces to

16 $$\begin{aligned} ax^3+bx^2+ax+d=0, \end{aligned}$$

where *a*, *b*, *d* are given by Eq. (12).

The (complex) roots of such an equation can be given explicitly by means of Cardano’s formula:

17 $$\begin{aligned} x^*= -\frac{b}{3a}+\root 3 \of {-\frac{q}{2}+\sqrt{\frac{q^2}{4}+\frac{p^3}{27}}} +\root 3 \of {-\frac{q}{2}-\sqrt{\frac{q^2}{4}+\frac{p^3}{27}}}, \end{aligned}$$

where

18 $$\begin{aligned} p=1-\frac{b^2}{3a^2},\;\;q=\frac{d}{a}-\frac{b}{3a}+\frac{2b^3}{27a^3}. \end{aligned}$$

It is well known that such a formula gives one, two or three real solutions, according to whether

$$\begin{aligned} \frac{q^2}{4}+\frac{p^3}{27}>0, \quad \frac{q^2}{4}+\frac{p^3}{27}=0, \quad \frac{q^2}{4}+\frac{p^3}{27}<0. \end{aligned}$$

In particular, if one has $p>0$, i.e., $3a^2-b^2>0$, (17) yields a unique real solution.

If, however,

19 $$\begin{aligned} 0<\alpha<-\delta<1, \qquad \gamma >0, \qquad 0<-\eta<\mu , \qquad 0<\beta <1-\gamma \mu , \end{aligned}$$

it follows that $a=\gamma \mu +1-\beta >0$, $b=(\beta -1)\delta -\gamma \eta >0$, $d=(\beta -1)(\alpha +\delta )-\gamma \eta >0$, $a-b=(1-\beta )(1+\delta )+\gamma (\eta +\mu )>0$ and therefore $3a^2-b^2>a^2-b^2=(a-b)(a+b)>0$. Hence, under assumption (19) Theorem 3.3 predicts system (5) to have a unique stable stationary point $(x^*,y^*)$, with $y^*=\frac{\mu x^*-\eta }{\beta -1}$ and $x^*$ given by Eq. (17). Moreover (by the positivity of the coefficients *a*, *b*, *d*), one has that $x^*<0$.

#### Example 3.5

For $n>2$ even and $\beta =1$, system (5) has, however, a unique stationary point $(x^*,y^*)$ given by

20 $$\begin{aligned} {\left\{ \begin{array}{ll} x^*=\frac{\eta }{\mu } \\ y^*=\frac{x^*-\alpha f_{n}(x^*)-\delta }{\gamma }, \end{array}\right. } \end{aligned}$$

which is stable if $0<\alpha <\frac{2}{n}$ and $0<\gamma \mu <1-\frac{\alpha n}{2}$.

In fact, by Proposition 2.1 (iii), one has $-\frac{n}{2}<f'_n(x^*)<\frac{n}{2}$. As a consequence, the Jacobian

$$\begin{aligned} \begin{bmatrix} \alpha f'_n(x) &{} \gamma \\ -\mu &{} 1 \end{bmatrix} \end{aligned}$$

has the properties

- $$\det (J)=\alpha f'_n(x^*)+\gamma \mu<\frac{\alpha n}{2}+\gamma \mu <1$$,
- $$1- Tr(J)+\det (J)=\gamma \mu >0$$,
- $$1+ Tr(J)+\det (J)=2(1+\alpha f'_n(x))+\gamma \mu>2\bigl ( 1-\frac{\alpha n}{2}\bigr )+\gamma \mu >0$$,

which, according to Theorem 3.3, proves the stability of $(x^*,y^*)$.

### Case $n=+\infty$

In this limiting case, we have

$$\begin{aligned} f_{\infty }(x) :=\lim _{n\,even, \,n\rightarrow +\infty } f_{n}(x)= {\left\{ \begin{array}{ll} 0 \qquad \text { if }\;|x|>1 \\ 1 \qquad \text { if }\;|x|<1 \\ \frac{1}{2} \qquad { if}\;|x|=1, \end{array}\right. } \end{aligned}$$

i.e., $f_{\infty }(x) = \Pi \bigl (\frac{x}{2}\bigr )$, where $\Pi$ represents the rectangle function. If $\alpha >0, \;\; 0<\beta<1, \;\; 0<\gamma \mu <1$, for stationary points, using $y=\frac{\mu x-\eta }{\beta -1}$ (see Eq. 10), we have $x=\frac{\gamma \eta +(1-\beta )(\delta +\alpha f_{\infty }(x))}{\gamma \mu +1-\beta }=:\Lambda +\nu f_{\infty }(x)$, where

$$\begin{aligned} \Lambda =\frac{\gamma \eta +(1-\beta )\delta }{\gamma \mu -\beta +1}, \qquad \nu =\frac{\alpha (1-\beta )}{\gamma \mu -\beta +1}, \quad \text {with} \; \nu >0. \end{aligned}$$

Since $f_{\infty }\in \{0,\frac{1}{2},1 \}$, the stationary points of system (5) satisfy one of the expressions

21 $$\begin{aligned} x^*_1=\Lambda , \quad x^*_2=\Lambda +\nu , \quad x^*_3=\Lambda +\frac{\nu }{2} \qquad \biggl (y^*_i=\frac{\eta -\mu x^*_i}{1-\beta } \quad ,i=1,2,3\biggr ). \end{aligned}$$

$(x^*_1,y^*_1)$ is therefore stationary if and only if (’iff’) $f_\infty (x^*_1)=0$, i.e. iff $|x^*_1|=|\Lambda | >1$; $(x^*_2,y^*_2)$ is stationary iff $f_\infty (x^*_2) = 1$, i.e. iff $|x^*_2|=|\Lambda + \nu | <1$; $(x^*_3,y^*_3)$ is stationary iff $f_\infty (x^*_3) = 1/2$, i.e. iff $|x^*_3|=|\Lambda + \nu /2| =1$.

Using $(\Lambda , \nu )$ as the pair of parameters, $(x^*_1, y^*_1)$ is stationary iff $(\Lambda , \nu )$ satisfies $\Lambda < -1$ or $\Lambda >1$ (half-planes); $(x^*_2, y^*_2)$ is stationary iff $(\Lambda , \nu )$ satisfies $-1< \Lambda + \nu < 1$ (stripe); $(x^*_3, y^*_3)$ is stationary iff $(\Lambda , \nu )$ lays on the lines $\lambda + \nu /2=-1$ or $\Lambda + \nu /2 =1$ (line).

**Figure 1:**
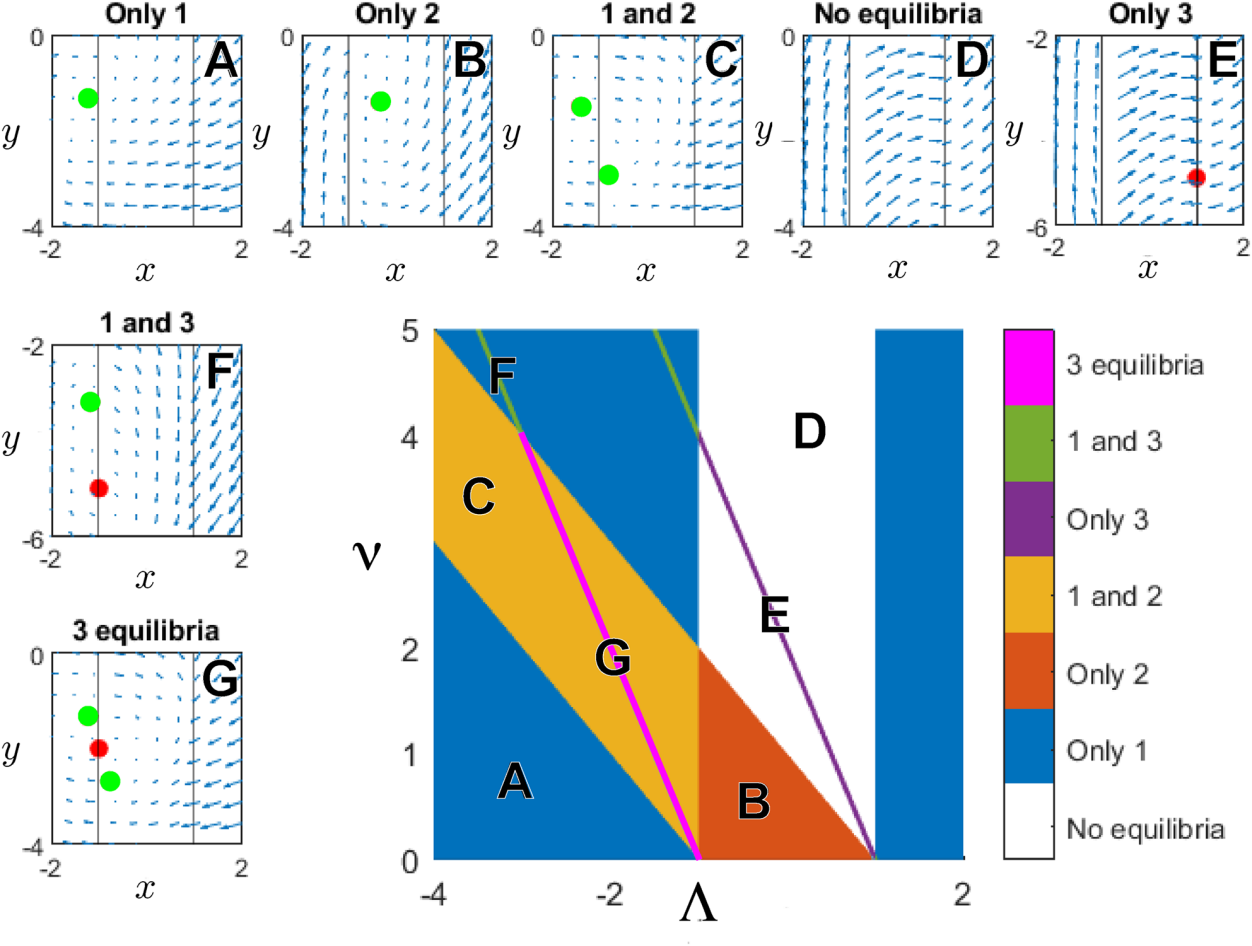
Center panel: Stationary states of system (5) in the $(\Lambda ,\nu )$ plane. Small figures: Exemplary illustrations using vector fields on the $\{x,y\}$ state-space with locations of stable fixed-points (green) and unstable fixed-points (red) indicated.

Thus, depending on the values of $(\Lambda , \nu )$, we obtain 0, 1, 2, 3 stationary points. By stability properties, the half plane $\nu >0$ divides into regions $A, B, \ldots , G$ (see the central rectangle of Fig. 1). The stationary points $(x^*_1, y^*_1)$ and $(x^*_2, y^*_2)$ will always be stable: The Jacobian matrix

$$\begin{aligned} J= \begin{bmatrix} 0 &{} \gamma \\ -\mu &{} \beta \end{bmatrix} \end{aligned}$$

satisfies Eq. (9), since one has $\det (J) = \gamma \mu <1 \text{ and } 1 \pm Tr(J) + \det (J) = 1\pm \beta + \gamma \mu >0$. The stability of the point $(x^*_3,y^*_3)$ can, however, not be determined by linearization, since in its neighbourhood the dynamics are discontinuous. Using numerical simulations we found that the stationary points at $x^*_3= \pm 1$ are unstable. We thus have

A. one stable stationary point $(x^*_1, y^*_1)$ if $\Lambda < -1$ and $\nu \le -1-\Lambda$, or if $\Lambda <-1$ ,   $\nu \ge 1-\Lambda$ and $\Lambda + \nu /2 \ne \pm 1$, or when $\Lambda >1$ and $\Lambda + \nu /2 \ne \pm 1$;
B. one stable stationary point $(x^{*}_{2}, y^{*}_{2})$ if $-1\le \Lambda \le 1$ and $-1-\Lambda<\nu <1-\Lambda$;
C. two stable stationary points, $(x^*_1, y^*_1)$ and $(x^*_2, y^*_2)$, if $\Lambda < -1$, $-1-\Lambda< \nu < 1-\Lambda$ and $\Lambda + \nu /2 \ne - 1$;
D. no stationary point if $-1\le \Lambda \le 1$, $1-\Lambda \le \nu$ and $\Lambda + \nu /2 \ne \pm 1$;
E. one unstable stationary point $(x^*_3, y^*_3)$ if $-1\le \Lambda \le 1$, and $\Lambda + \nu /2 = \pm 1$;
F. two stationary points, $(x^*_1, y^*_1)$ (stable) and $(x^*_3, y^*_3)$ (unstable), if $\Lambda < -1$, or $\Lambda >1$ and $\Lambda + \nu /2 = \pm 1$;
G. three stationary points, $(x^*_1, y^*_1)$ (stable), $(x^*_2, y^*_2)$ (stable) and $(x^*_3,y^*_3)$ (unstable), if $\Lambda < -1$, $-1-\Lambda< \nu < 1-\Lambda$ and $\Lambda + \nu /2 = - 1$.

For $n\rightarrow \infty$, we therefore observe seven possible scenarios, depending on the number of stationary points obtained.

## Numerical investigations of the theoretical model

Theoretical results are generally valid only locally around the stationary state and therefore provide little information regarding basins of attraction involved. Moreover, they fail to reveal what happens if stationary states become unstable. “Stationary states and their stability” section provides sufficient, but not necessary, conditions that yield only conservative predictions of the dynamics to be expected, and also leave the dependence on the order *n* of the response function *f* unresolved.

To remedy this weakness, we complement our theoretical study with a numerical analysis, where the state space is explored starting from random initial conditions by means of $10^3$ simulated paths of 1200 iteration steps each, using different choices of *n*. The numerical tests suggest that the partition into seven areas obtained from asymptotic *n* is still valid for *n* non-asymptotic. Together with the central asymptotic $(\Lambda ,\nu )$ plane partition of Fig. 1, vector-field sub-panels illustrate this finding, where our specifically chosen parameter values were

- A: $$\beta =0.8, \alpha =0.2, \delta =0.1, \gamma =1, \eta =-1, \mu =0.6$$;
- B: $$\beta =0.8, \alpha =0.1, \delta =0.1, \gamma =0.4, \eta =-1, \mu =2$$;
- C: $$\beta =0.8, \alpha =2, \delta =0.1, \gamma =1, \eta =-1, \mu =0.5$$;
- D: $$\beta =0.8, \alpha =4, \delta =0.1, \gamma =0.4, \eta =1, \mu =2$$;
- E: $$\beta =0.8, \alpha =5.8, \delta =0.1, \gamma =0.4, \eta =1, \mu =2$$;
- F: $$\beta =0.8, \alpha =1.8, \delta =0.1, \gamma =0.4, \eta =-3, \mu =2$$;
- G: $$\beta =0.8, \alpha =1.8, \delta =0.1, \gamma =1, \eta =-1, \mu =0.6$$.

More detailed numerical results are presented in the following subsections.

### Case $2<n<\infty$

**Figure 2:**
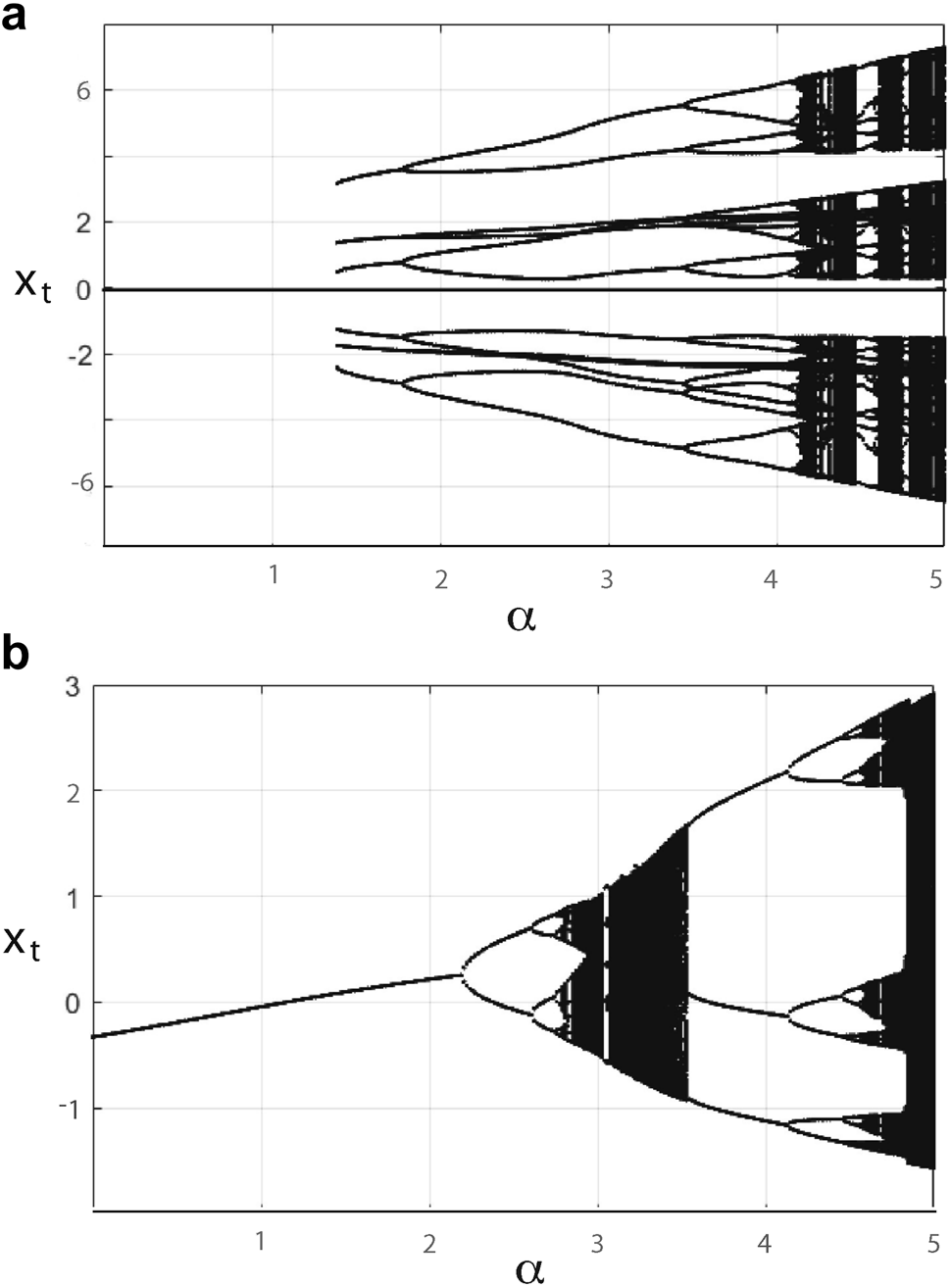
Asymptotic behaviour of system (5) (**a**) for $n=4, \beta =1, \delta =0, \gamma =1, \eta =-0.05, \mu =0.9$ and (**b**) for $n=2, \beta =0.99, \delta =-0.05, \gamma =1, \eta =-0.0011, \mu =0.0025$, for different values of $\alpha$. Last 200 steps from 1200 iterations from $10^3$ initial conditions are shown.

Figure 2a exhibits the behavior obtained for variable $\alpha$, where for $\alpha < 1/3$ the assumptions of Theorem 3.3 are satisfied. The simulations confirm that when $\beta =1$ there is always a stationary state with $x^* = \eta / \mu$, and that for all explored values of $\alpha$, this stationary state is stable. This does not contradict Theorem 3.3 and Example 3.5, since the latter represents a sufficient, but not a necessary, condition. For small $\alpha$, the stationary state (20) is the only attractor for $n\rightarrow \infty$; at $\alpha = 1.39$ the system becomes multistable, by saddle-node bifurcation giving birth to a stable period-6 cycle. Depending on the specificity of parameter $\alpha$, the system either tends to the stationary state (20) or is attracted by another periodic, or chaotic, orbit. Upon increasing $\alpha$, a Feigenbaum cascade^20, 21^ emerges, leading from periodic to chaotic behavior.

### Case $n=2$

Figure 2b collects the results obtained for $\alpha \ne - 0.05$ (thus either satisfying or violating the assumptions of Theorem 3.3 and Example 3.4, respectively). The simulations confirm the theoretical result that for small $\alpha$, the stationary point (17) is stable. As a sufficient condition, the theoretical threshold of $\alpha$ is, however, very conservative; in reality, the stationary state loses stability for $\alpha = 2.2$, due to a supercritical flip (or ’period-doubling’) bifurcation. At that level of $\alpha$, the stable stationary state is replaced by a stable cycle of period 2 that at $\alpha = 2.6$ loses its stability and is replaced by a stable cycle of period 4. These are the first steps of a flip-type or period doubling bifurcation cascade^20, 21^ that generally leads to a chaotic attractor dense of unstable periodic orbits. For $\alpha \in [3.55,4.13]$ the only attractor of the system is a period 3 limit cycle.

### Case $n\rightarrow \infty$

**Figure 3:**
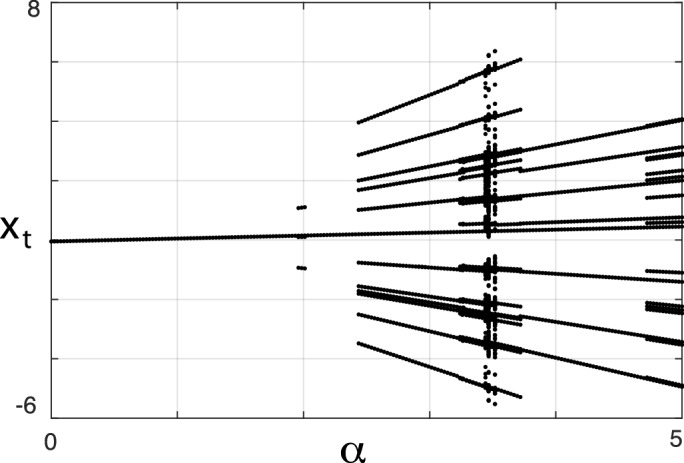
Asymptotic behavior of (5) for $n\rightarrow \infty , \beta =0.9, \delta =0, \gamma =1, \eta =-0.05, \mu =0.9$ and different values of $\alpha$. Last 200 of 1200 iterations of $10^3$ simulation runs.

Figure 3 reports the last 200 iterations (out of 1200 simulation steps) of $10^3$ simulated paths with $n\rightarrow \infty$, illustrating that the process always approaches a stable state. This not only confirms the arguments of “[Sec Sec5]” section; additionally, it exemplifies that the stationary state of this system does not need to be a simple period one: As $\alpha$ increases to $\alpha = 2.45$, a period-11 arises through to a saddle-node bifurcation and disappears through the same mechanism at $\alpha = 3.75$, whereas at $\alpha \ge 3.68$ a period-7 emerges from a saddle-node bifurcation of the loop, persisting up to $\alpha =6.1$ where stability is lost due to a supercritical period doubling bifurcation (bifurcation parameters and types were identified using the MatContM^22^ software).

This is the rough overview of the landscape in which the process when modeled by our approach will take place. It will emerge from our numerical investigations involving real-world data, that, as approaches with different orders *n* yield results of similar quality, the order *n* of function *f* cannot be fixed in a decisive manner (cf. Table 4 below).

## Android’s market position

**Figure 4:**
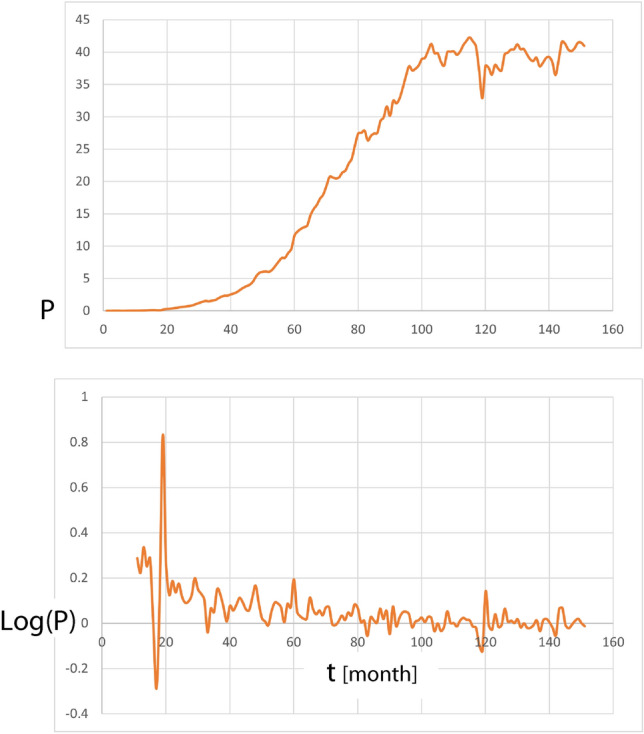
Evolution of Android’s market percentage P, top panel, and corresponding logarithmic changes Log(P), bottom panel, from January 2009 to July 2021 (monthly data).

We demonstrate in the following that our deterministic modeling captures Android’s market competition data. Over the last decade from January 2009 to July 2021, Android’s market percentage rose from 0 to 40.96%, cf. Fig. 4 (based on monthly retrieved StatCounter^23^ data). Table 1 exhibits the main statistical properties of this data, where skewness and excess kurtosis account for a heavily asymmetric dynamics with extreme deviations.

**Table 1:** First four central moments of the logarithmic market share development, from January 2009 to July 2021.

OS	$\mu _1$	$\mu _2$	$\mu _3$	$\mu _4$
Android	0.0512	0.1033	3.3299	26.6497

Such properties pose problems for modelling and forecasting. While outliers are generally seen as inconsistent observations^24^, in economics and finance, skewness and kurtosis are inherent characteristics, and extreme observations are common^25,26^. In univariate financial time series, innovations are assumed to be symmetric, so that outliers are more difficult to detect than in independent data, since a single outlier may affect subsequent observations^27^. When it comes to modelling and forecasting, the computational challenge is kurtosis maximisation. To solve that problem, a recent suggestion was to convert projections with maximal kurtosis in univariate financial time series, into an easier to solve eigenvalue problem^28^. However, kurtosis-based projection pursuit, aimed at removing excess kurtosis, suffers from a crucial drawback: Kurtosis may not be defined for relevant distributions (e.g., the Student-t distribution with 4 or less degrees of freedom; for non-normal multivariate distributions, the fourth cumulant may be a null matrix), so that kurtosis might not be an appropriate projection index^29^ (for the situation in emerging stock markets, see Ref.^30^). Our low-dimensional deterministic approach masters the computational challenge posed by the highly skewed time series with extreme kurtosis in a computationally cheap manner.

### Empirical parameter inference for forecasting and in-sample prediction

The empirical time series we use captures the monthly percentual change of Android’s market share. We first present modeling results, where we compare our low-dimensional deterministic approach based on Rulkov maps with an elaborate stochastic approach. We first demonstrate that the proposed deterministic approach is as good as the traditional stochastic one. The parameters yielding the optimal results permit us then to determine the location of the real-world data within our theoretical system landscape.

The first step in our analysis consists in calibrating the Rulkov approach. Calibration involves a nonlinear regression, performing a robust estimation via an iteratively re-weighted least squares algorithm^31^. At each iteration, based on the residuals from the previous iteration, the weights are recalculated, until they converge. To compare the results with those of the stochastic approach, the ARIMA-EGARCH approach was taken, with degrees, (*p*, *d*, *q*) and (*a*, *b*), respectively, chosen using the Bayesian information criterion (BIC) and the Akaike information criterion (AIC). In the following, we will denote the achieved ’optimal’ stochastic model by ARIMA-EGARCH*. To arrive at a two-dimensional representation, a slow component *y* is associated with the market dynamics data *x*, by means of performing an exponentially weighted moving average^32–34^ on *x*. Evaluations of the dynamic time warping distances confirm that the differences between the stochastic and the Rulkov approaches are at the level of white noise effects (see our Appendix for details).

#### Forecasting

**Figure 5:**
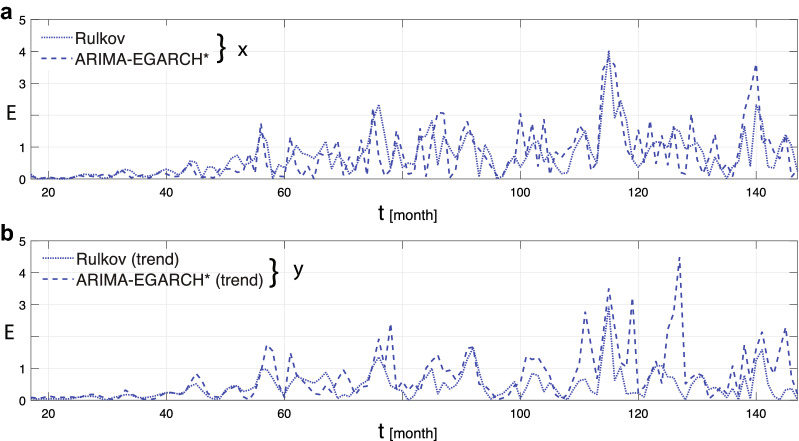
Absolute percentage forecast error *E*: deterministic versus stochastic approach, components *x* and *y* (Android market share data).

For a first strong demonstration of the strength of our deterministic approach, we consider forecasting. For this, we take the values $x_s, \; y_s$ known at time *s*, from which, with the help of (5), the values at times $t>s$ are calculated. Model parameters are calibrated over the rolling window $[s-m+1,s]$, where $m=12$ is chosen due to the monthly frequency of the data (in contrast to a calibration across the whole data set shown later). Using the mean absolute percentage error (MAPE)^35^ and the absolute prediction error as measures, the obtained forecasts are compared to those from the ARIMA-EGARCH* model (the latter based on a mean of $10^3$ iterations), see Table 2 and Fig. 5. The two approaches evidence similar fits both for the process and its trend, confirming that the deterministic model performs at least as well as the stochastic one.

**Table 2:** Forecast errors: deterministic versus stochastic approach.

	Rul. map state var.	MAPE Rul. map	MAPE ARIMA-GARCH*
Android TS	*x*	0.0849	0.0815
	*y*	0.0563	0.0655

#### In-sample prediction

As an even stronger second argument for the strength of our approach are the results obtained by calibrating Rulkov and ARIMA-EGARCH*, respectively, over the whole time series, cf. Table 3.

**Table 3:** Estimated Rulkov parameters for components *x* and *y*.

Parameters	$\alpha$	$\gamma$	$\delta$	$\beta$	$\mu$	$\eta$	*n*
Android TS	− 0.093582	− 1.2302	0.097117	− 0.6695	− 0.32162	0.002575	2

**Figure 6:**
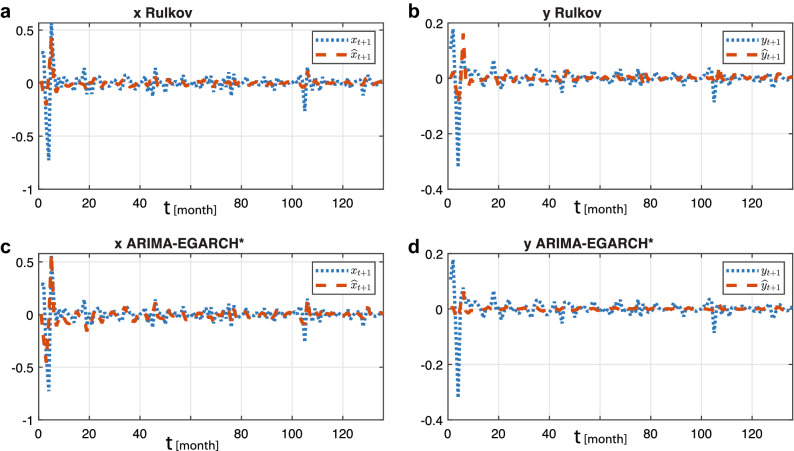
Modeling comparison: (**a**) Android time series variable *x* (blue), versus Rulkov state variable ${\hat{x}}$ (red); (**b**) Android time series variable *y* (blue), versus Rulkov state variable ${\hat{y}}$ (red). (**c**) Android time series variable *x* (blue), vs. ARIMA-EGARCH* state variable ${\hat{y}}$ (red); (**d**) Android time series variable *y* (blue), versus Rulkov state variable ${\hat{y}}$ (red).

This setting poses a major modeling challenge when volatility is high, or if the system changes from one regime of behavior to another. The quality of the modeling results reported in Fig. 6, is corroborated in Table 4 ($n=2$-panel) by the ratio between the relative mean absolute error RMAE and the normalized root-mean square error (NRMSE). From visual inspection and error data, it is evident that both approaches capture the market dynamics equally well.

#### Stability of the empirical state

Table 3 reports the parameters that we have obtained from the analysis of the in-sample calibrated time series. Albeit the particular case of a negative value of $\alpha$ was neither included in the presented model discussion nor in our numerical investigations, numerical investigations reveal that the parameters of the empirical state satisfy the conditions (19) of Example 3.4, so that the motion around the equilibrium state $(-0.0246,0.0005)$ should be globally stable. Across the time scales embraced by our empirical data, indeed a deterministic convergence to the mentioned fixed-point is observed on intermediate time scales, on which over shorter time scales a stochastic component seems to act as a ’disturber’. Table 4 confirms our earlier made observation that the order *n* of the reactivity plays a minor role in the modeling.

**Table 4:** Error of the deterministic versus the stochastic approach.

Rul. map spec.	ARIMA-EGARCH* parameters	Time series	Rul. map state var.	RMAE (Rul. map/ARIMA-EGARCH*)	NRMSE Rul. map	NRMSE ARIMA-EGARCH*
n = 2	(1,1,2),(2,1)	Android	*x*	1.0473	0.0742	0.0704
	(1,1,2),(2,1)		*y*	0.9233	0.0673	0.0710
n = 4	(1,1,2),(2,1)	Android	*x*	1.0597	0.0939	0.0704
	(1,1,2),(2,1)		*y*	1.0263	0.0701	0.0710
n = 30	(1,1,2),(2,1)	Android	*x*	1.0598	0.0940	0.0704
	(1,1,2),(2,1)		*y*	1.0569	0.0722	0.0710

To date, whether financial data should preferentially be seen as deterministic or as stochastic processes is still unsettled. Some of the available data appear, however, to exhibit a substantial chaotic component^36–38^. Measures that indicate such a property are a positive maximal Lyapunov exponent^39–43^ (leaving open potential randomness); similarly, large values of the approximate entropies indicate that fluctuations over a time series are unpredictable^44,45^ (e.g., a regular alternation of 1 and 0 yields the value 0.0022 compared to around 0.63 for a random sequence of 0 and 1). As additional means, correlation dimensions measure the fractal dimension D of the space occupied by the data points^43,46,47^. For one-dimensional time series, D is directly related to the Hurst exponent^48^ H by $D= 2 - H$^49,50^ (for stock markets, H has been shown to be around 0.5-0.6^51,52^). Finally, the normalized ’spectral entropy’ variant SE^53^ of Shannon’s entropy^54^ measures the average level of ”information” in a random variable^55–57^. Table 5 exhibits the values of the above-mentioned descriptors of the real-time series of Android market share, demonstrating consistency with the results from the Rulkov approach. While these results, indeed, seem to suggest a chaotic component in the data, most of the numerical methods used are, unfortunately, hampered by the lack of reaching a reliable saturation regime upon using variable embedding dimensions, and thus should not be used to umpire between deterministic chaotic vs. stochastic market data.

**Table 5:** Chaotic descriptors: Android time series versus Rulkov map modelling.

Max. Lyap. Exp.		Approx. Entr.		Corr. Dim.		Hurst Exp.	
Andr. TS	Rul. map	Andr. TS	Rul. map	Andr. TS	Rul. map	Andr. TS	Rul. map
0.4115	0.4424	0.4831	0.5014	3.0845	2.8599	0.5899	0.6686

**Figure 7:**
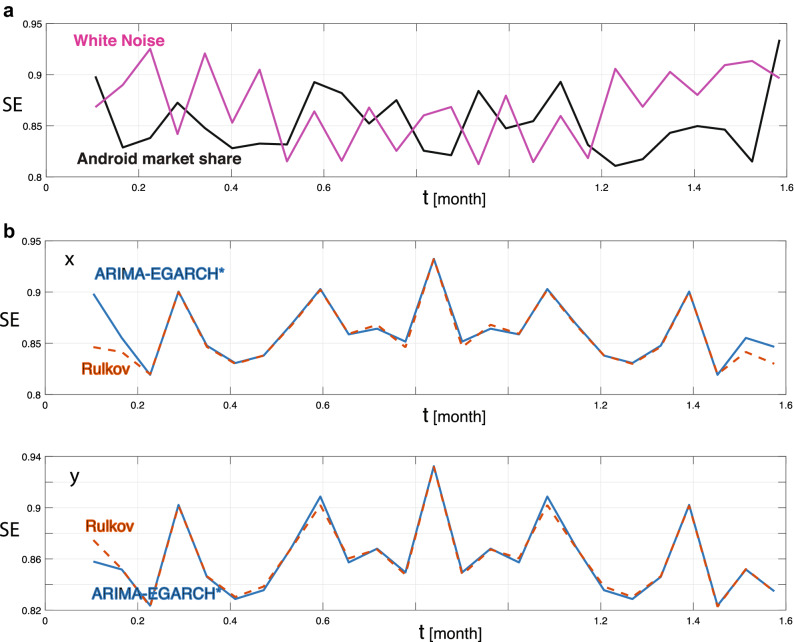
Spectral entropy SE (**a**) of the log-changes of Android’s market share (black) versus that of a white noise signal (magenta) exhibiting similar entropy levels; (**b**) of the stochastic (blue, continuous line) vs. the deterministic (orange, dashed line) calibrated approaches, for *x*- (top) and for *y*-variable (bottom). The almost perfect overlap of the two aligns well with the real-world data shown in (**a**).

Figure 7a compares the entropy for the log-changes of Android’s market share versus the entropy of a random white noise process, confirming that the information content of both signals is high, which can be taken as an indication of either chaos or randomness being present in the data. Figure 7b compares the spectral entropy SE of the ARIMA-EGARCH* with that of the Rulkov approach, showing an almost complete overlap of results.

**Figure 8:**
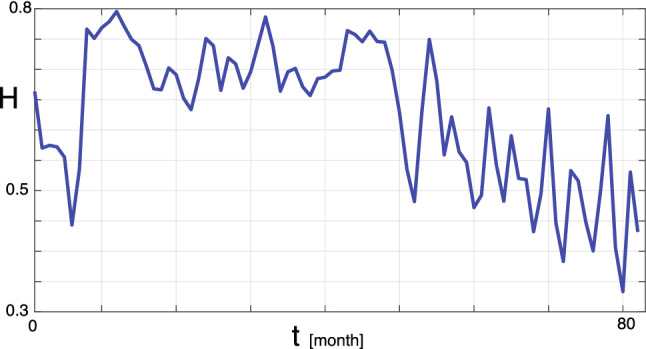
Evolution of the Hurst exponent H of the logarithmic Android market share changes (rolling windows).

The temporal evolution of the Hurst exponent shown in Fig. 8 confirms the possibility of a low-dimensional chaotic component in the data.

## Conclusions

We explored the advantages that a low-dimensional deterministic approach may have when applied to traditionally considered stochastic data. Comparisons of in-sample calibration, forecasting and stability analysis of the two deterministic with the stochastic approach, have revealed an example of a commonly exclusively stochastic considered data that hosts a substantial ingredient of deterministic features. For our real-world computer operating systems market share data, our findings indicate stochasticity prevails on the shortest scale and determinism on the intermediate scale. On larger time scales (above one year) the dynamics appear to change again (details not included). Given a desired time-scale, excess entropies, a recently developed tool for comparing models with data^58^ will provide more details on the accurateness of the modeling of the data. We suspect that beyond the specific data investigated, such a characterization may be a generic feature of a large class of real-world complex multicomponent systems, where our advocated deterministic approach offers insight into otherwise hidden structures that underlie the production of individually stochastic events. Our detailed analysis focused on the model’s dependence on the parameter $\alpha$ expressing the system’s responsiveness on short time scales. Upon a change of this parameter, stationary states may lose their stability, give way to a Feigenbaum cascade and to other bifurcation phenomena. In this way, our deterministic modeling reveals a strongly multistable nature underlying the generation of individual events, where the identified equilibria may be targets or avoided, by taking appropriate control measures^59,60^. Thus, in addition to providing short-term predictions of reliability comparable to that obtained from the stochastic view, the deterministic approach offers an overview of the landscape of potential behaviours, that upon a change of externally accessible parameters may be monitored from outside by means of external guidance.

From a more global perspective, our investigations reveal strong similarities between local scale market behavior (as pursued here) with that at the more global level (such as the financial stress index), and neuronal firing behavior from various contexts. The embracing system category may, independently of the application specificity, bear substantial importance as a class of theoretical study.

## Data Availability

The data that support the findings of this study are available from the corresponding author upon reasonable request.
